# Supplementary material for: Generation and maintenance of the circularized multimeric IS26-associated translocatable unit encoding multidrug resistance
Source: Commun Biol. 2024 May 18;7:597. doi: 10.1038/s42003-024-06312-4 (PMC11102541; doi:10.1038/s42003-024-06312-4)
Supplement: Supplementary file 2 — Supplementary information [file 42003_2024_6312_MOESM2_ESM.pdf]

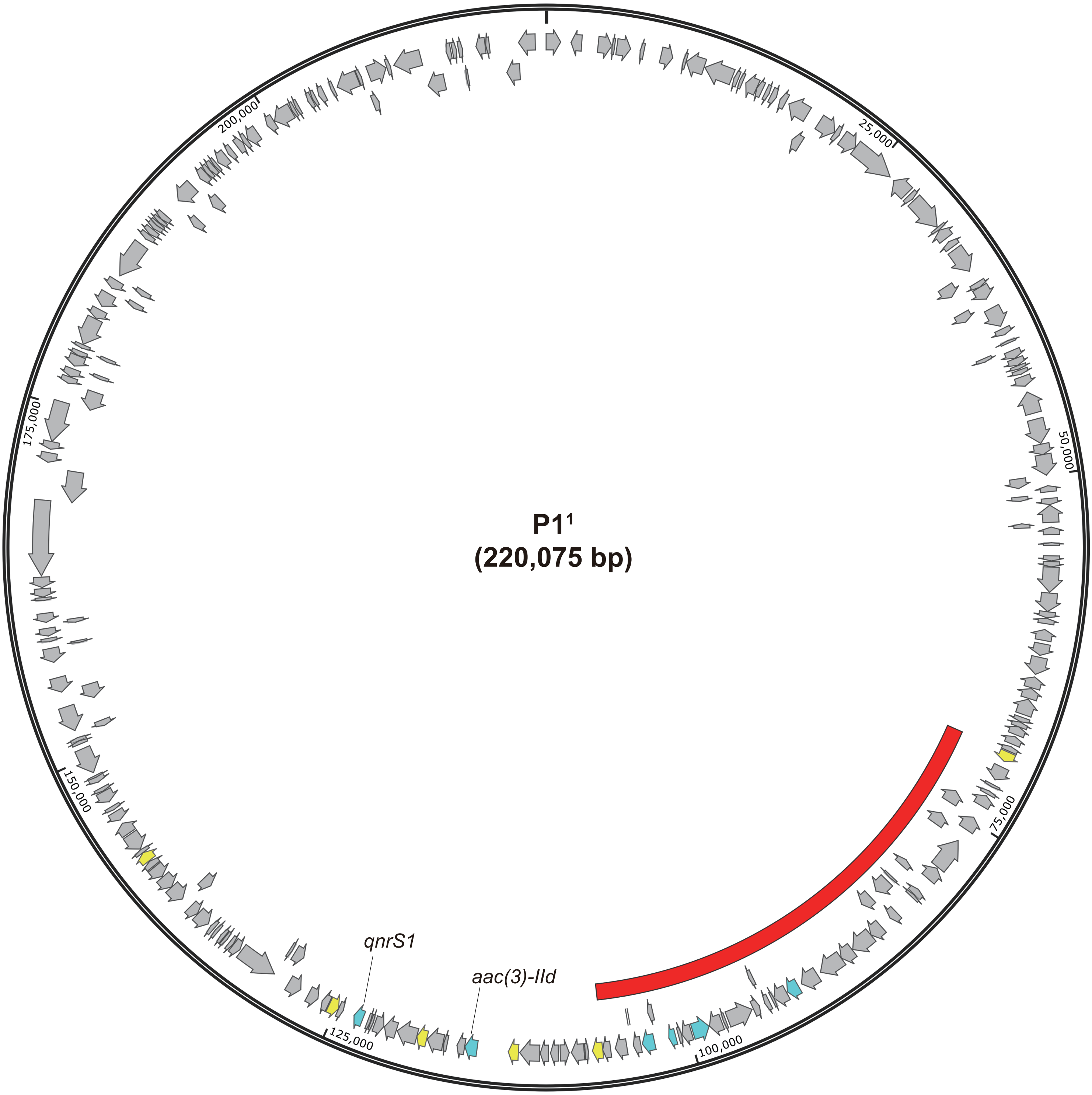

Supplementary Fig. 1: Circular map of P1[1]. Arrows indicate the positions and orientations of each protein-coding sequence. Intact IS*26* TPases and AMR genes are indicated in yellow and blue, respectively. The pP2 region is indicated by a red line. See the main text for the AMR genes in pP2.

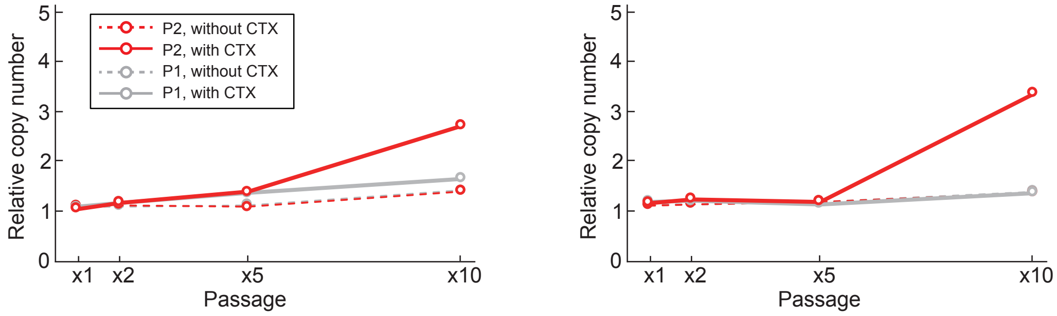

Supplementary Fig. 2: Changes in the copy numbers of P1 and P2 by subculturing KpWEA1 in the presence or absence of sub-MICs of CTX. Two KpWEA1 colonies different from the one examined in the main text (Fig. 4b) were subjected to x10 passages as described in Fig. 4a.

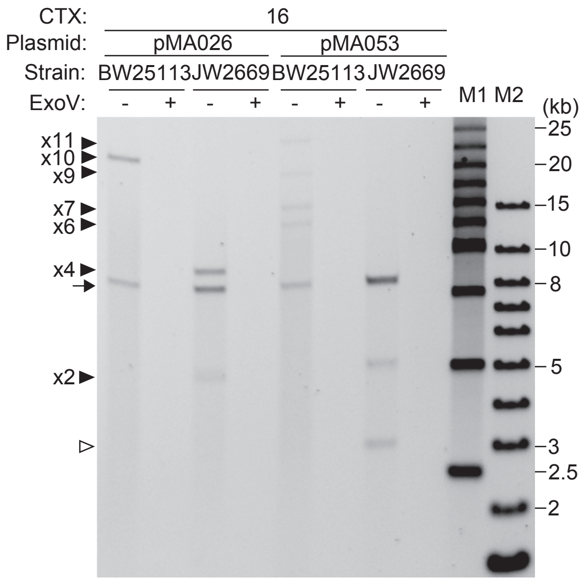

Supplementary Fig. 3: The FIGE profiles of BamHI-digested fragments derived from pMA026 and pMA053 derivatives in the presence or absence of ExoV. The extrachromosomal DNA (100 ng) was extracted from the pMA026- or pMA053-introduced BW25113 and JW2669 cells passaged five times in the presence of 16 µg/mL CTX and was digested with BamHI in the presence or absence of ExoV. The arrow and open arrowhead indicate fragments derived from the vector plasmid sequence and the mini-PCTn where no amplification of mini-pP2 occurred, respectively. Filled arrowheads indicate the fragments derived from the mini-PCTn where the mini-pP2 was repeated 2, 4, 6, 7, 9, 10, and 11 times (x2, x4, x6, x7, x9, x10, and x11). M1 and M2 are the same size markers as those used in Fig. 7b.

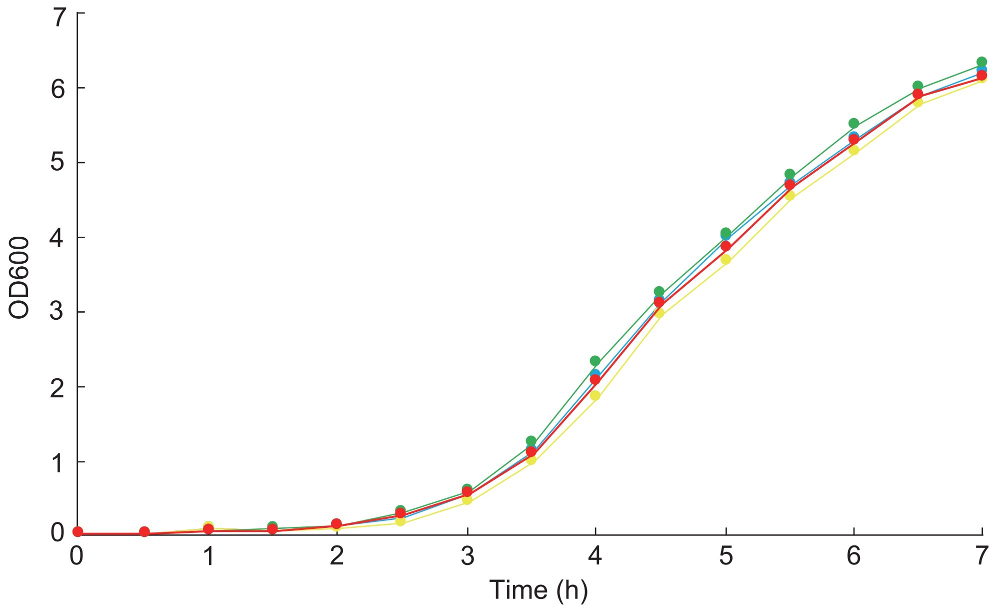

Supplementary Fig. 4: The growth curve of *E. coli* BW25113 containing P1[1] (blue), P1[2] (yellow), P1[3] (green), or P1[4] (red). Among the P1[2], P1[3] or P1[4] transformants analysed in Fig. 6d, those found to contain the highest numbers of P2 copies were examined. The cells precultured in LB at 37°C overnight were inoculated into fresh LB at the optimal density at 600 nm (OD600) of 0.01 and cultured at 37°C with shaking at 150 rpm. The OD600 of each culture was measured every 30 min for 7 h.

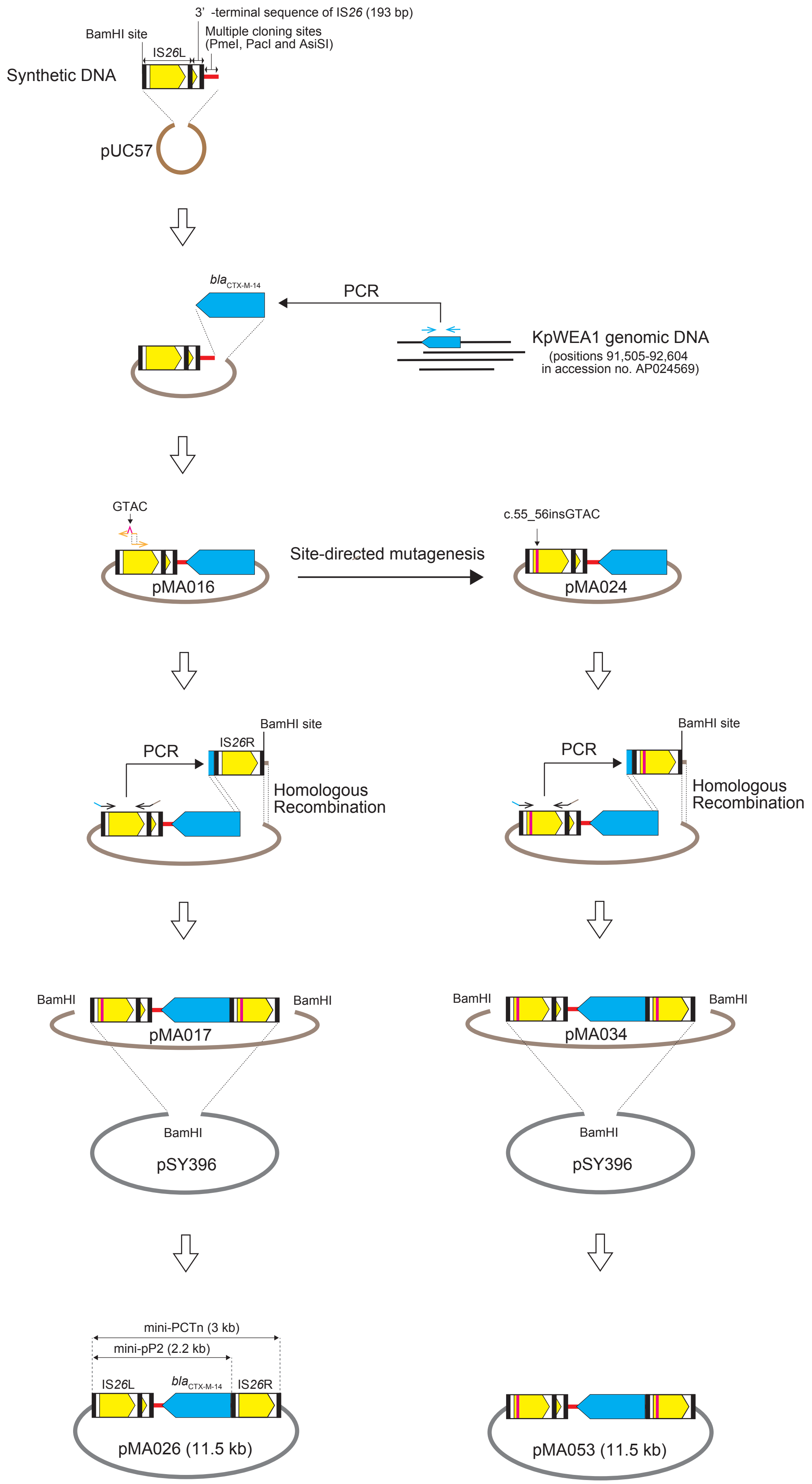

Supplementary Fig. 5: The processes to construct pMA026 and pMA053.

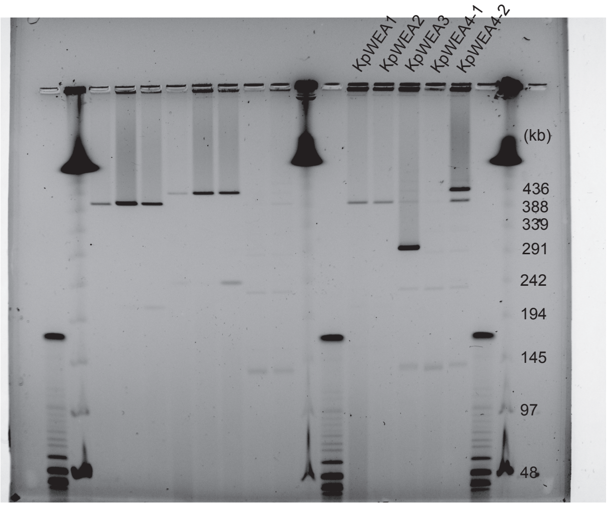

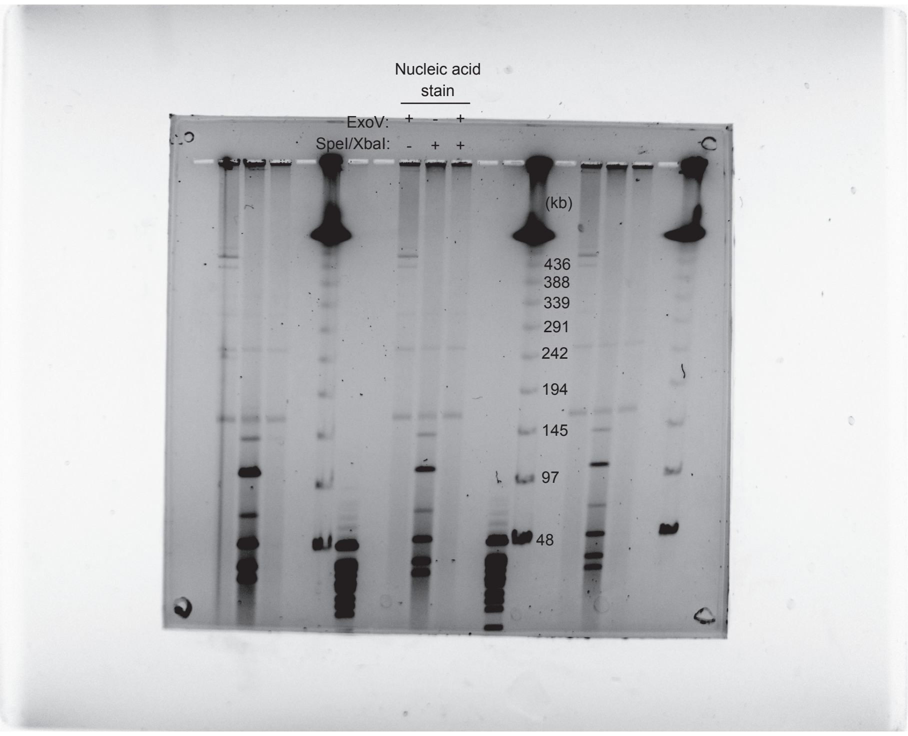

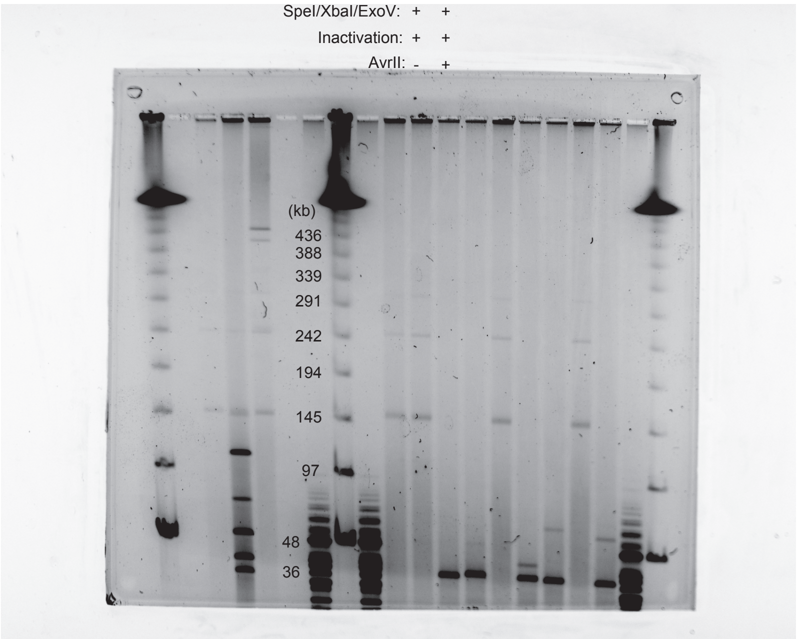

Supplementary Fig. 6: Uncropped PFGE images (Fig. 3).

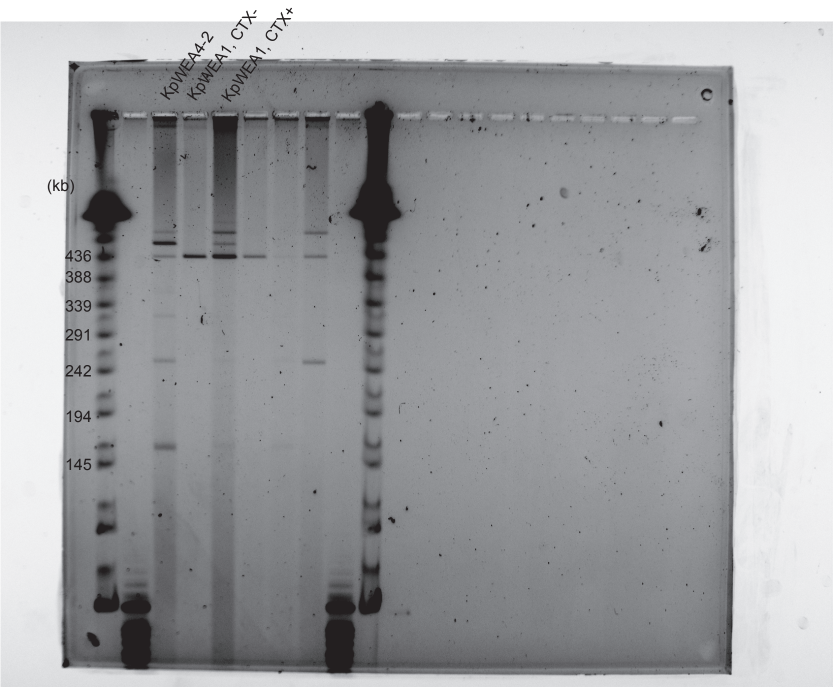

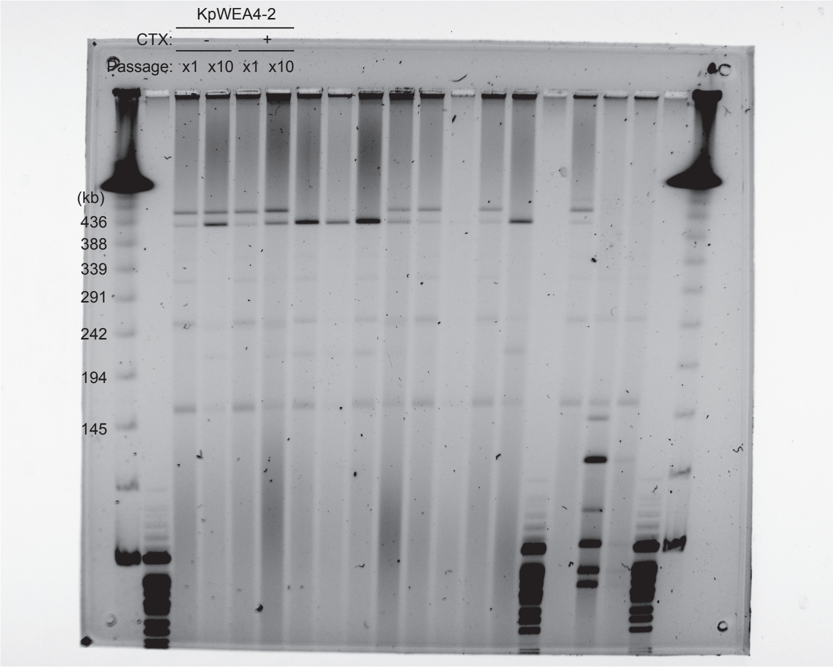

Supplementary Fig. 7: Uncropped PFGE images (Fig. 4).

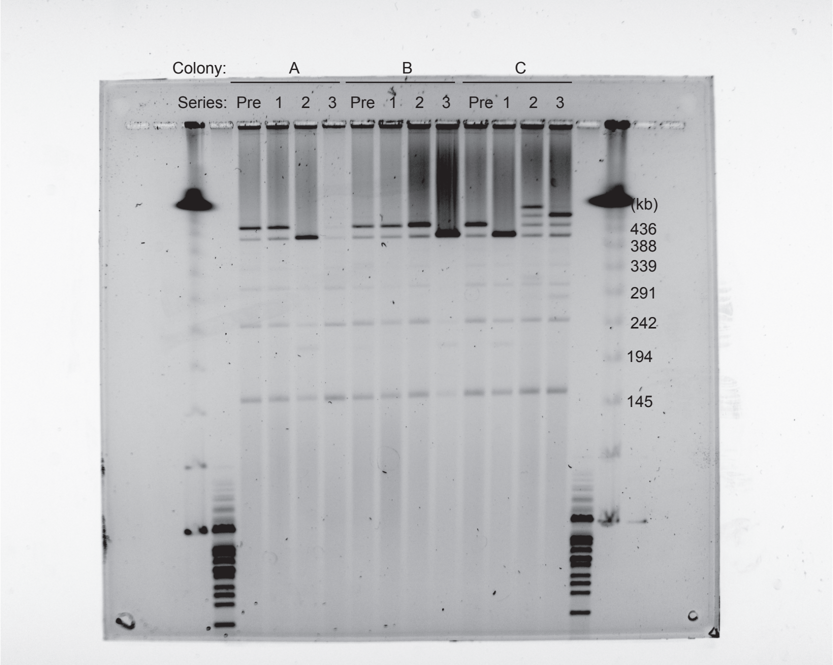

Supplementary Fig. 8: An uncropped PFGE image (Fig. 5).

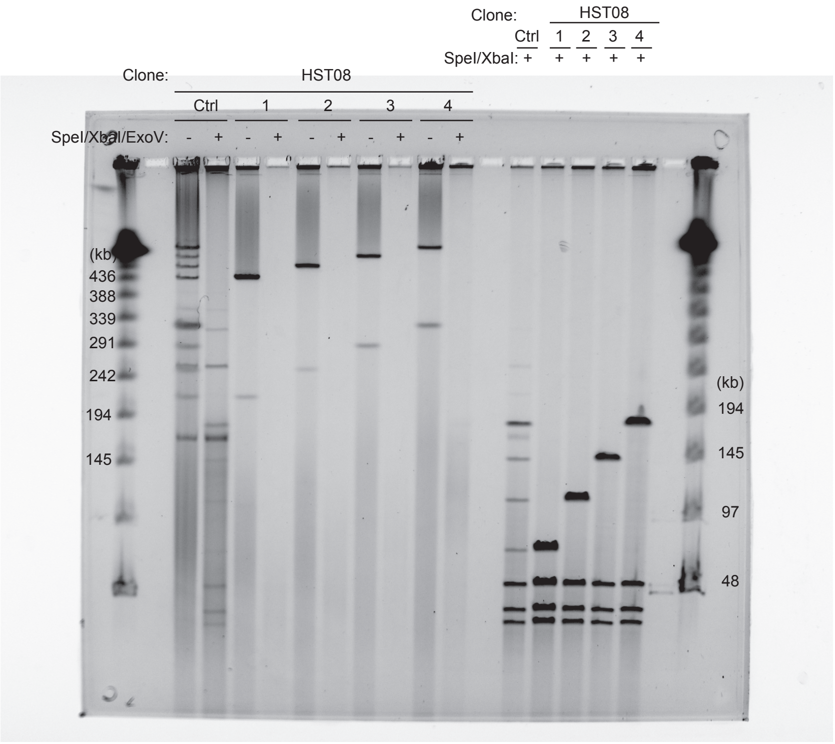

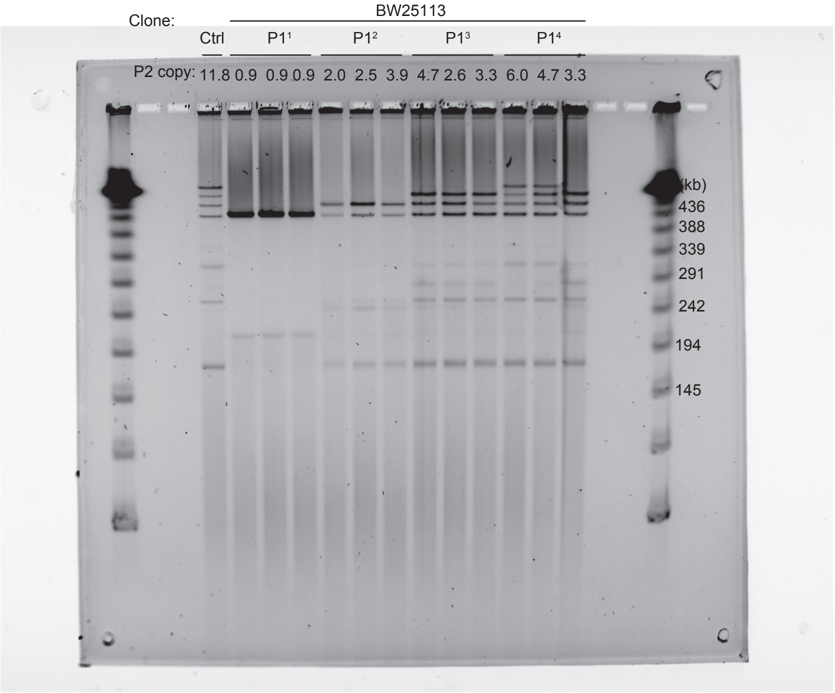

Supplementary Fig. 9: Uncropped PFGE images (Fig. 6).

Supplementary Table 1: Primers and probes used in this study.

Target gene and genome	Sense primer (5' to 3')	Antisense primer (5' to 3')	Probe (5' to 3')
Chromosome of *K. pneumoniae*	aggcatcaacttctacggc	gttcgctttaccctgacc	attgagaaagcgggtgcctggtat
Chromosome of *E. coli*	cgctcggctatttcacatact	ggtttctggcgttaaaccaatc	tttctacctttctggagcgtctggc
P1	gataagacaaccggcgaag	ttcagagttgggacgaagag	acggtactggtgctgttgacatcc
P2	caacgctgccgtttgataag	attccagcgtcacctttagc	cggtctatgtggaaggcgctca
sopA (pSY396)	gtgaagcagcggatttagttgg	gtcctcgaatttccatatccgg	atcagagatgccgagaaagcaggg
bla$_{CTX-M-14}$ for ddPCR	tgcagtacagcgacaatacc	agtgcgatccagacgaaac	acaaattgattgcccagctcggtg
bla$_{CTX-M-14}$ for hybridization probe	agaagatttgtcgtatctcgcg	cggttacggtctgcatattcac	-
IS*26* mutagenesis	ttctgtgggccgtacgtacgctggta ctgcaaatacggcatc	gtacggcccacagaatgatgtcac	-
IS*26*R cloning (insert)	atccacagagcaacaggcactgttg caaatagtcggtgg	tgcatctagatatcggatccggcactgtt gcaaagttagcgatg	-
IS*26*R cloning (vector)	tgttgctctgtggataacttg	cgatatctagatgcattcgc	-

Supplementary Table 2: Minimal inhibitory concentrations (MICs) of *E. coli* BW25113 electroporated each P1 derivative.

Strain	P1 electroporated	Relative P2 copy number	MIC to cefotaxime (μg/mL)
BW25113	P1[1]	0.9	16
		0.9	32
		0.9	32
	P1[2]	2.0	128
		2.5	128
		3.9	128
	P1[3]	4.7	128
		2.6	128
		3.3	128
	P1[4]	6.0	128
		4.7	128
		3.3	128
